# Investigating related factors with mortality rate in patients with postoperative meningitis: One longitudinal follow up study in Iran

**Published:** 2018-04-04

**Authors:** Arezoo Chouhdari, Kaveh Ebrahimzadeh, Omidvar Rezaei, Mohammad Samadian, Giv Sharifi, Mohammadreza Hajiesmaeili

**Affiliations:** 1 Skull Base Research Center, Loghman Hakim Medical Center, Shahid Beheshti University of Medical Sciences, Tehran, Iran; 2 Clinical Research Development Center, Loghman Hakim Hospital, Shahid Beheshti University of Medical Sciences, Tehran, Iran; 3 Anesthesiology Research Center, Loghman Hakim Medical Center, Shahid Beheshti University of Medical Sciences, Tehran, Iran

**Keywords:** Incidence, Mortality Rate, Related Factor, Postoperative Complications, Meningitis

## Abstract

**Background:** Postoperative meningitis (POM) is the most dreaded cause of morbidity and mortality in neurosurgery patients. This study aimed to identify incidence and mortality rate as well as related factors to outcome in patients with POM.

**Methods:** This descriptive longitudinal study conducted on patients with POM in duration of 2 years. Incidence and mortality rates as well as related factors were studied.

**Results:** The incidence and mortality rates of POM was 8.9% and 50%, respectively. There were statistically significant association between male gender, as well as having mechanical ventilation, and death outcome in univariable analysis. In addition, in multivariable logistic regression analysis, length of intensive care unit (ICU) stay of more than 7 days [Odds ratio (OR): 1.2, confidence interval of 95% (95%CI): 1.02-6.2), mechanical ventilation (OR: 1.1, 95%CI: 1.05-5.1], positive cerebrospinal fluid (CSF) culture (OR: 2.4, 95%CI: 1.9-4.08) were predicting factors to death outcome. Finally, we found an inverse relationship between survival function and length of ICU stay in patients with POM.

**Conclusion:** According to the high rates of incidence and mortality due to POM in this study, preventive studies to decrease this dreaded cause of morbidity and mortality in neurosurgery patients should be the planned.

## Introduction

Postoperative meningitis (POM) is an uncommon (0.3-8.1 percent) but life-threatening complication of intracranial surgery.^1^^-^^3^ This complication is fatal with high morbidity and mortality rates of 20 to 50%.^4^^,^^5^ Moreover, POM is associated with prolonged hospitalization, multiple surgeries, broad-spectrum antibiotic treatment, and increase in total cost of illness.^6^^-^^7^

Several risk factors have been known, such as postoperative cerebrospinal fluid (CSF) leakage, CSF shunts, concurrent incision infection, long-time operation over 4 hours, and emergency surgery.^8^^,^^9^ Other studies have reported some risk factors like a set of a foreign body duration surgery, previous neurosurgical infection, the absence of antibiotic prophylaxis, interventions involving nasal sinuses, and previous radiation therapy.^8^^-^^10^ In Kourbeti, et al. study, the incidence of post-neurosurgery meningitis reported about 4.8%, and CSF cultures were positive for microbial growth in 100% of the cases.^4^

Despite the discovery of many factors associated with the incidence of POM, research on the factors associated with mortality is very limited. So in this study, we investigated the incidence and mortality rates, as well as related factors in patients with POM in Loghman Hakim hospital, Tehran, Iran, as a tertiary university hospital, during the years 2015-2017.

## Materials and Methods

This descriptive longitudinal study was conducted from 21 March 2015, through March 21, 2017. All 425 patients who underwent neurosurgery (open or closed) were evaluated for detection of POM duration 30 days in neurosurgery intensive care unit (ICU) of Loghman Hakim hospital, a tertiary university hospital. For diagnose of POM the criteria of Centers for Disease Control and Prevention (CDC)^9^ were used. 

Basic characteristics of patients were collected. Incidence and mortality rates, as well as related factor associated to mortality rate, were evaluated. This study was approved by the ethical committee of Shahid Beheshti University of Medical Sciences, Tehran. 

To report the descriptive results, the mean and standard deviation (SD), and for quantitative data, number and percent were used. For data analysis, chi-square and Fisher’s exact tests, as well as, independent t and Mann-Whitney U tests, were used. We used logistic regression to show predicting factors for the outcome of patients with POM. The survival curve was plotted, too. A significant level of P < 0.05 was considered for all tests. Statistical analysis was done using SPSS software (version 19, SPSS Inc., Chicago, IL, USA).

## Results

In this descriptive longitudinal study, the incidence rate of POM according to other criteria was 8.9% (n = 34). Background characteristics and clinical and paraclinical findings of patients with POM are visible in table 1. 

**Table 1:** Basic characteristics of patients with postoperative meningitis (POM)

**Variable**		**Mean ± SD**	
Age		46.41 ± 16.94	
Length of hospital stay before surgery (days)			2.26 ± 2.95
Duration of surgery (hours)			5.49 ± 2.56
Time between surgery and POM diagnosis (days)			3.67 ± 2.95
Mechanical Ventilation (days)		10.04 ± 10.76	
CSF lactate (mmol/l)		55.57 ± 32.68	
Length of ICU stay (days)			11.05 ± 7.66
Length of hospital stay (days)		24.44 ± 15.44	
Sex[n (%)]	Men		18 (52.5)
	Women		16 (47.1)
Diagnosis[n (%)]	Brain tumor		15 (44.1)
	Adenoma		5 (14.7)
	Hydrocephalus		7 (20.6)
	Cerebrovascular disease		4 (11.8)
	Trauma		2 (5.9)
	Colloid cyst		1 (2.9)
Type of surgery[n (%)]	Open		32 (94.1)
	Close		2 (5.9)
Surgery status[n (%)]	Emergency		11 (32.4)
	Elective		23 (67.6)
Mechanical Ventilation[n (%)]	Yes		23 (67.6)
	No		11 (32.4)
Prophylactic Antibiotics[n (%)]	Yes		34 (100)
	No		0 (0)
Postoperative ventricular drain[n (%)] or shunt	Yes		8 (23.5)
	No		26 (76.5)
Headache[n (%)]	Yes		23 (65.7)
	No		12 (34.3)
Fever[n (%)]	Yes		18 (52.5)
	No		16 (47.1)
GCS[n (%)]	3-8		16 (47.1)
	9-12		9 (26.5)
	13-15		9 (26.5)
CSF culture[n (%)]	Yes		5 (14.7)
	Acinetobacter		4 (11.8)
	Staphylococcus		1 (2.9)
	No		29 (85.3)
Outcome[n (%)]	Death		17 (50.0)
	Mild disability		2 (5.9)
	Recovery		15 (44.1)

SD: Standard deviation; POM: Postoperative meningitis; CSF: Cerebrospinal fluid; ICU: Intensive care unit; GCS: Glasgow coma scale

**Table 2:** Results of multiple logistic regression analysis to predict outcome of death in patients with postoperative meningitis (POM)

**Variables**	**Surveyed**	**Reference**	**P**	**OR (95% CI)**
Length of ICU stay	7 days <	7 days ≥	0.04	1.2 (1.02-6.20)
Mechanical ventilation	Yes	No	0.03	1.1 (1.05-5.10)
Positive CSF culture	Yes	No	0.01	2.4 (1.90-4.08)

OR: Odds ratio; 95% CI: Confidence interval of 95%; ICU: Intensive care unit; CSF: Cerebrospinal fluid

In univariable analysis, there was not any statistically significant association between the outcome (death and alive with or without disability) with age (P = 0.08), reoperation (P = 0.50), ventricular drain or shunt (P = 0.90), length of hospital stay before surgery of more than 7 days (P = 0.30), duration of surgery of more than 4 hours (P = 0.20), length of ICU stay (P = 0.05), length of hospital stay (P = 0.06), CSF lactate (P = 0.07), CSF leakage (P = 0.60), positive CSF culture (P = 0.10), or surgical site infection (P = 0.50). Only there was a statistical significant association between patient outcome with male gender (P = 0.01), and with having mechanical ventilation (P < 0.01). 

In multivariable logistic regression analysis, length of ICU stay of more than 7 days (P = 0.04), mechanical ventilation (P = 0.03), and positive CSF culture (P = 0.01) were predicting factors for death outcome (Table 2).

**Figure 1:**
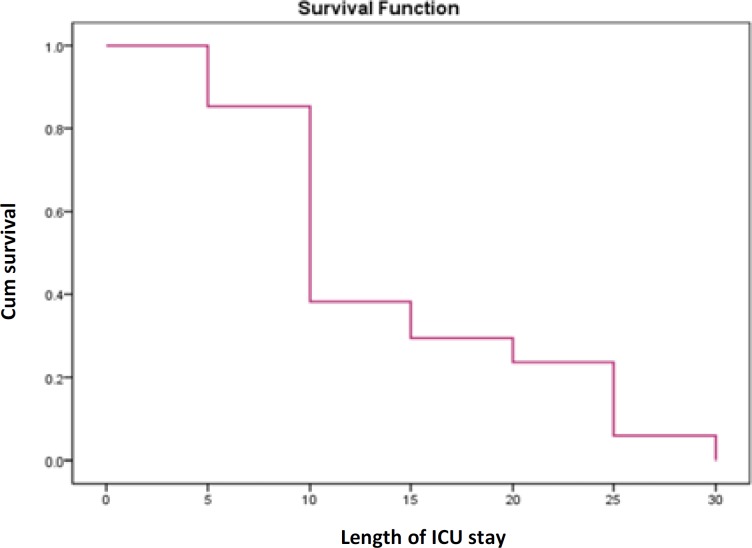
Survival curve in patients with postoperative meningitis (POM) in intensive care unit (ICU)

In this study, we found an inverse relationship between patients’ survival and length of ICU stay (Figure 1).

## Discussion

In this survey, despite only 5 positive CSF cultures, an incidence rate of POM according to other criteria was 8.9%; 4 cases were acinetobacter, and the other was staphylococcus. Kourbeti, et al. study showed the incidence of POM as 5% that most common organisms were gram-positive cocci.^5^ In Dashti, et al. survey, the most common organisms causing meningitis were non-lactose fermenting Gram-negative bacillus followed by Pseudomonas and Klebsiella species.^10^ In one of the largest neurosurgical studies, the incidence of meningitis after neurosurgical procedures was less than 1%.^1^ This low incidence of POM (< 1%) may relate to prescription of antibiotics prophylaxis. But, in our study, despite the administration of antibiotic prophylaxis in all patients, incidence rate of POM was high. Another study indicated that antibiotic prophylaxis reduced incision infections from 8.8% down to 4.6% (P < 0.001), but did not prevent meningitis; 1.63% in patients without antibiotic prophylaxis, and 1.5% in those who received prophylaxis antibiotic.^8^

Our study showed a significant association between male gender and death outcome in univariable analysis (P = 0.01). In Kono, et al. survey, male gender was a risk factor for POM, too (P = 0.02, OR: 3.97, 95% CI: 1.21-13.03).^3^

Moreover, in current research, length of ICU stays of more than 7 days), mechanical ventilation, and positive CSF culture were predictors for death outcome. In Rezaei, et al.^6^ and Yadegarynia, et al.^7^ studies, long-time operation of over 4 hours, and length of hospital stay before surgery of over 7 days were predicting factors for POM onset. 

Although only 5 positive CSF culture were found due to preoperative prophylaxis antibiotics administration, it was anticipator for death outcome in patients with POM. Another study showed the CSF leakage, diabetes mellitus, and male gender were associated with an increased mortality rate of POM.^1^

## Conclusion

Despite prescription prophylaxis antibiotics in all neurosurgeries, incidence and mortality rate of POM was high. Therefore, it is important to conduct more research on risk factors influence POM.

## Notes:

**How to cite this article:** Chouhdari A, Ebrahimzadeh K, Rezaei O, Samadian M, Sharifi G, Hajiesmaeili M. Investigating related factors with mortality rate in patients with postoperative meningitis: One longitudinal follow up study in Iran. Iran J Neurol 2018; 17(2): 82-5.
